# Quetiapine-associated leucopenia and thrombocytopenia: a case report

**DOI:** 10.1186/s12888-015-0495-9

**Published:** 2015-05-07

**Authors:** Kuang-Yuan Fan, Wen-Yin Chen, Ming-Chyi Huang

**Affiliations:** Department of Psychiatry, Taipei City Psychiatric Center, Taipei City Hospital, 309, Song-De Road, Taipei, Taiwan; Department of Psychiatry, School of Medicine, Taipei Medical University, Taipei, Taiwan

**Keywords:** Quetiapine, Leucopenia, Thrombocytopenia

## Abstract

**Background:**

There have been few reports regarding quetiapine-associated hematological effects other than white-blood-cell alteration. We present the first reported Han-Chinese case that developed leucopenia and thrombocytopenia after taking quetiapine.

**Case presentation:**

We present a case of a person with a bipolar I disorder who experienced leucopenia and thrombocytopenia after taking 400 mg/day of quetiapine and 1000 mg/day of valproic acid for three and one-half months.

The hematological toxicity abated upon the discontinuation of both drugs. However, due to the intolerable side effects of the replaced antipsychotic (haloperidol), and according to the patient’s preference, we prescribed quetiapine and valproic acid again. There was a recurrence of leucopenia and a decreased platelet count by the sixth day. The adverse effects disappeared soon after we discontinued quetiapine, while keeping valproic acid treatment.

**Conclusion:**

Quetiapine-associated leucopenia and thrombocytopenia seems reversible but possibly fatal. Therefore, clinical practitioners should be aware of this adverse reaction.

## Background

Quetiapine is a commonly-prescribed atypical antipsychotic drug used worldwide for schizophrenia and mood disorders. It is a dibenzothiazepine derivative related to clozapine in chemical structure and pharmacological profile [1,2].

Several cases of quetiapine-associated hematologic side effects have been reported. Leucopenia (a white blood cell count below 4.0 × 10^3^/μL) develops in approximately one percent of the subjects treated with quetiapine, and it is resolved upon discontinuation [1]. In addition, some reports indicate neutropenia (an absolute neutrophil count below 1.5 × 10^3^/μL) and agranulocytosis (an absolute neutrophil count below 500/μL) may emerge when using quetiapine [1].

There have been fewer case reports addressing quetiapine-associated hematological effect of combined alteration of white-blood-cells and platelets. We could find only two reports in previous literature reporting quetiapine-associated leucopenia and thrombocytopenia (a platelet count below 150 × 10^3^/μL) [2,3]. We will present a case study of a person who developed leucopenia and thrombocytopenia after taking quetiapine.

## Case presentation

Mr. A, a twenty-three-year-old man, was diagnosed as having a bipolar I disorder at the age of nineteen due to the manifestations of manic symptoms with psychotic features. He had been admitted six times in various hospitals for the management of recurrent manic episodes. He had successively taken lithium, valproic acid, and carbamazepine, though with poor drug compliance after his hospital discharge. He did not have a history of systemic or hematologic diseases.

After taking 400 mg/day of quetiapine and 1000 mg/day of valproic acid for four weeks, with no other psychotropic agents, he was transferred to our rehabilitation ward for occupational training. On the first day of admission, routine blood tests revealed a white blood cell (WBC) count of 4.37 × 10^3^/μL, an absolute neutrophil count (ANC) of 1.56 × 10^3^/μL, a red blood cell (RBC) count of 4.63 × 10^6^/μL, a hemoglobin (Hb) count of 15.7 g/dL, and a platelet count of 149 × 10^3^/μL.

Unfortunately, after a total of three and one-half months with the above medications, he developed a sudden onset of fever and upper respiratory tract infection (Day 0). Two days later (Day 2), his leucopenia, neutropenia, and thrombocytopenia counts were noted as follows: WBC 1.99 × 10^3^/μL, ANC 860 /μL, platelet 81 × 10^3^/μL, while his RBC and Hb remained in the normal ranges. We stopped using valproic acid on Day 2 and closely monitored the clinical conditions. However, the fever persisted; and the laboratory data showed agranulocytosis with thrombocytopenia (WBC 1.63 × 10^3^/μL; ANC 500/μL; platelet 76 × 10^3^/μL) on Day 4. Therefore, we immediately discontinued quetiapine. His clinical condition stabilized on Day 6, with the lab data showing the following: WBC 2.84 × 10^3^/uL, ANC 1,107/μL, and platelet 91 × 10^3^/μL. On day 8, there was an obvious improvement of the hematological profile (WBC 7.92 × 10^3^/μL; ANC 5,726/μL; platelet 171 × 10^3^/μL).

On Day 9, due to an acute worsening of manic and psychotic symptoms, we prescribed 10 mg/day of haloperidol. However, we noticed severe and intolerable extra-pyramidal side effects. Considering the patient’s preference, 200 mg/day of quetiapine and 500 mg/day of valproic acid were administered on Day 18 and were titrated up to 400 mg/day and 1000 mg/day, respectively on Day 20. With a cautious follow-up of the hematological data, we noted the following: leucopenia (WBC 3.12 × 10^3^/μL), a decreased neutrophil count (ANC 1,719/μL), and thrombocytopenia (136 × 10^3^/μL) again on Day 24 without clinical symptoms. On Day 26, we discontinued quetiapine and shifted the medication to 4 mg/day of risperidone, while maintaining the valproic acid treatment. On Day 30, we observed normal white-blood-cell and neutrophil count (WBC 7.22 × 10^3^/μL, ANC 5,595/μL), and a platelet count closer to normal range (platelet 149 × 10^3^/μL). The patient has not been administered quetiapine since then, and the hematological follow-up examinations have all revealed normal results thereafter.

We have presented a case of a man with a bipolar I disorder who developed leucopenia, neutropenia, and thrombocytopenia after being administered quetiapine and valproic acid. The hematological abnormalities were resolved upon discontinuation of both drugs but re-emerged following the reuse of them. However, the abnormalities abated again after we stopped quetiapine but continued using valproic acid. The temporal relationship between drug exposure and laboratory data suggests direct quetiapine toxicity. In our case, the Naranjo adverse drug-reaction probability scale was seven, which suggested a probable causality.

We could find only two reports in previous literature reporting quetiapine-associated leucopenia and thrombocytopenia [2,3]. Both of them were published case report, in which the adverse reactions were described in detail. They reported hematological toxicity after 600 mg/day of quetiapine were administered for five weeks [2] and 400 mg/day for four years [3]. These adverse effects, similar to our patient’s, were all resolved upon discontinuation of quetiapine and the investigations for other etiological causes all showed no remarkable findings. However, neither of them stated the reuse of quetiapine as our case. There has been only one case report describing quetiapine-associated pancytopenia (50 mg/day for 3 weeks) [4]. These reports showed that the duration of quetiapine use before the onset of hematological adverse effects might be between several weeks to several years. As in our case, the duration was approximately three and one-half months, which was compatible with previous reports.

However, we cannot completely exclude the contributing effect of valproic acid. Two case reports also showed that neutropenia might be induced by a combined use of quetiapine and valproic acid [5,6]. It has been suggested that the co-medication of valproic acid would increase the plasma level of quetiapine by seventy-seven percent because of the inhibiting effect on cytochrome P450 3A4 [6]. Nevertheless, we believe quetiapine played a crucial role in the hematological toxicity of our case since we observed a rapid restoration after the cessation of its use. Similarly, another case report also showed that thrombocytopenia occurred after co-administration of quetiapine and valproic acid but still persisted despite valproic acid discontinuation, suggesting a unique propensity of quetiapine to trigger hematological toxicity [7].

The mechanisms underlying the toxicity remained unclear. Given a resemblance of the chemical structure and similar pharmacological profile, it has been speculated that quetiapine could cause bone-marrow depression similar to clozapine [2]. Both drugs are characterized by high 5HT_2_-relative-to-DA_2_ receptor affinity and virtually no affinity for muscarinic receptors [2]. Olanzapine is another antipsychotic drug which is related to quetiapine and clozapine in chemical structure and pharmacological profile. The rarer adverse effect of the combination of leucopenia and thrombocytopenia has also been reported in patients administered olanzapine [8]. Other mechanisms, including immune-mediated toxicity, induction of free radicals, and the formation of an intermediate nitrenium ion, have also been proposed to explain the phenomenon [3].

According to our case report, we do not have adequate information to conclude whether this phenomenon was dose-related or idiosyncratic. A previous case report stated that quetiapine-induced leucopenia might be a possible dose-dependent phenomenon [9]. According to the experience of clozapine, both dose-dependent toxicity and dose-independent hypersensitivity are possible mechanisms of clozapine-induced blood dyscrasia, which might show some similarity to quetiapine [10]. Further studies are needed to investigate this phenomenon.

We presented the first case in Han-Chinese of quetiapine-associated leucopenia and thrombocytopenia. Another unique aspect of our case is the reuse of quetiapine in the treatment course, which hasn’t been mentioned in previous case reports. Nevertheless, the contributing effects from the co-administered valproic acid cannot be neglected. We hope our case report can help in accumulating evidence and enhance further investigation to find out clinical or biological factors related to this side effect.

## Conclusion

Quetiapine-associated leucopenia and thrombocytopenia seem reversible but possibly fatal. Although it has not been a clinical routine yet for a regular check-up of the complete blood cell count in patients administered quetiapine, clinical practitioners should be aware of this adverse reaction.

## Consent

Written, informed consent was obtained from the patient for publication of this case report and any accompanying image. A copy of the written consent is available for review by the editor of this journal.

## Abbreviations

WBC: White blood cell
ANC: Absolute neutrophil count
RBC: Red blood cell
Hb: Hemoglobin
